# Cysteine-Rich Protective Antigen (CyRPA) as promising blood-stage candidate protein for inclusion in a malaria subunit vaccine

**DOI:** 10.1186/1475-2875-11-S1-P30

**Published:** 2012-10-15

**Authors:** Paola Favuzza, Anita M Dreyer, Sergio Wittlin, Hugues Matile, Gerd Pluschke

**Affiliations:** 1 Department of Medical Parasitology & Infection Biology, Swiss Tropical and Public Health Institute, Basel, Switzerland; 2 Pharma Research Basel, F. Hoffmann-La Roche Ltd., Basel, Switzerland

## Background

The development of an effective malaria vaccine is recognized as one of the most promising approaches that would provide a cost-effective intervention for addition to the currently available malaria control measures. Since the fully annotated *P. falciparum* genome has become available in 2002, reverse vaccinology represents a new opportunity to identify novel malaria vaccine candidate antigens. Screening of predicted *P. falciparum* open reading frames for proteins that could elicit parasite-inhibitory antibodies has led to the identification of the Cysteine-Rich Protective Antigen (CyRPA) as promising blood-stage candidate protein for inclusion in a malaria subunit vaccine.

## Materials and methods

On the basis of available genome-wide transcriptomic and proteomic information generated since 2002, we have selected uncharacterized ORFs for evaluation of their potential as vaccine candidate antigens. To generate tools for the characterization of candidate antigens, we have developed a cell-based approach for monoclonal antibody production: (I) generation of stably transfected mammalian cells, expressing high levels of target antigen on their surface in a native conformation; (II) immunisation of mice with transfected cells; (III) hybridoma cell generation by screening with the transfectants [1]. Stage-specific expression of CyRPA in schizonts and free merozoites was shown by Western blot analysis and confirmed by indirect immunofluorescence staining of synchronized blood-stage parasites. Generated anti-CyRPA mAbs showed parasite growth inhibitory activity due to inhibition of merozoite invasion. The *in vivo* growth inhibition was assessed by passive immunisation experiments in *P. falciparum* infected NOD-scid *IL2Rγ^null^* mice engrafted with human erythrocytes [2]. To demonstrate that growth inhibitory anti-CyRPA Abs can be induced by active immunization, CyRPA was recombinantly expressed as secreted protein in mammalian cells and directly purified from culture supernatant. Vaccine-induced polyclonal anti-rec_CyRPA Abs showed that the antigen is highly immunogenic in mice. Monoclonal antibodies against rec_CyRPA have been raised and are currently being characterized.

## Results and conclusions

Our data on localization, stage-specific expression pattern, and functional assays suggest a role of CyRPA in erythrocyte invasion by the merozoite. Importantly, CyRPA elicits Abs that inhibit merozoite invasion *in vitro* and *in vivo.* Thus, CyRPA represents a promising malaria blood-stage vaccine candidate antigen. It fulfills three key criteria applied to select asexual blood-stage antigens as vaccine candidates: (I) the protein is conserved; (II) Abs against the antigen inhibit parasite growth *in vitro* and (III) are protective in animal models. We expect that characterization of further parasite proteins with this strategy will identify additional vaccine candidate antigens from the extracellular stages of *P. falciparum.* This will extend the panel of vaccine antigens for incorporation into an effective multivalent, multi-stage malaria subunit vaccine.
